# Inhibition of Glycoprotein VI Clustering by Collagen as a Mechanism of Inhibiting Collagen-Induced Platelet Responses: The Example of Losartan

**DOI:** 10.1371/journal.pone.0128744

**Published:** 2015-06-08

**Authors:** Peng Jiang, Stéphane Loyau, Maria Tchitchinadze, Jacques Ropers, Guillaume Jondeau, Martine Jandrot-Perrus

**Affiliations:** 1 Inserm, UMR_S1148, Paris, France; 2 Univ Paris Diderot, Sorbonne Paris Cité, UMR_S1148, Paris, France; 3 APHP- CNMR Syndrome de Marfan et apparentés, Service de Cardiologie, Hôpital Bichat, Paris, France; 4 Unité de Recherche Clinique, Hôpital Ambroise Paré—UFR Médecine Paris-Ile-de-France-Ouest, Université Versailles St-Quentin, Boulogne, France; Medical Faculty, Ludwig Maximilians University Munich, GERMANY

## Abstract

Exposure of platelets to collagen triggers the formation of a platelet clot. Pharmacological agents capable of inhibiting platelet activation by collagen are thus of potential therapeutic interest. Thrombus formation is initiated by the interaction of the GPIb-V-IX complex with collagen-bound vWF, while GPVI interaction with collagen triggers platelet activation that is reinforced by ADP and thromboxane A2. Losartan is an angiotensin II (Ang II) type I receptor (AT1R) antagonist proposed to have an antiplatelet activity via the inhibition of both the thromboxane A2 (TXA2) receptor (TP) and the glycoprotein VI (GPVI). Here, we characterized *in vitro* the effects of losartan at different doses on platelet responses: losartan inhibited platelet aggregation and secretion induced by 1 μg.mL^-1^ and 10 μg.mL^-1^ of collagen with an IC50 of ~ 6 μM. Losartan inhibited platelet responses induced by the GPVI specific collagen related peptide but not by the α2β1 specific peptide. However, losartan did not inhibit the binding of recombinant GPVI to collagen, which is not in favor of a simple competition. Indeed, the clustering of GPVI observed in flow cytometry and using the Duolink methodology, was inhibited by losartan. The impact of a therapeutic dose of losartan (100 mg/day) on platelet responses was analyzed *ex vivo* in a double blind study. No statistically significant differences were observed between losartan-treated (n=25) and non-treated (n=30) patients in terms of collagen and U46619-induced platelet activation. These data indicate that in treated patients, losartan does not achieve a measurable antiplatelet effect but provide the proof of concept that inhibiting collagen-induced GPVI clustering is of pharmacological interest to obtain an antithrombotic efficacy.

**Trial Registration:**

ClinicalTrials.gov NCT00763893

## Introduction

After damage to the vessel wall, the exposure of the subendothelial matrix proteins to the blood results in platelet adhesion, activation and aggregation. Together with the activation of the coagulation cascade, this leads to the formation of a clot that seals the injury site. This process is critical for stopping bleeding but may also contribute to thrombotic disorders (ie. arterial thrombosis) [1]. At high shear rates platelets are captured via the interaction between the platelet glycoprotein (GP)Ib-V-IX complex and the von Willebrand factor (vWF) [2]. Collagen, the most thrombogenic subendothelial matrix component, triggers directly the adhesion of platelets to the vessel wall via its interaction with the α2β1 integrin. This facilitates the activation of platelets by collagen through GPVI, which predominantly mediates collagen-induced platelet responses [1, 3]. GPVI is a platelet membrane glycoprotein, a member of the immunoglobulin (Ig) superfamily, containing two extracellular Ig-like domains, a transmembrane region and a short cytoplasmic tail [4, 5]. It forms a non-covalent complex with the FcR γ-chain that drives signaling downstream of GPVI. Upon clustering of GPVI by collagen, the tyrosine residues of the FcR γ-chain immunoreceptor tyrosine-based activation motif (ITAM) are phosphorylated by the Src family kinases, Fyn and Lyn, initiating an intracellular signaling cascade and subsequent degranulation, activation and aggregation [6]. Blockade of GPVI interaction with collagen by either an anti-GPVI antibody or the fusion protein of GPVI-Fc blocks platelet adhesion, aggregation and thrombus formation [7, 8]. In addition, GPVI deficiency in humans and mice does not cause serious bleeding tendency [9–11]. Thus GPVI is considered to be a promising antiplatelet target [12].

Losartan, an AT1R antagonist, has been reported to exert an antiplatelet activity besides its antihypertensive effect. First, Liu et al. demonstrated that losartan is a competitive antagonist for TP and inhibits the aggregation of human platelets triggered by the TXA2 analogue U46619 [13]. This effect was also confirmed in spontaneously hypertensive rats [14]. Next, losartan was reported to inhibit platelet adhesion to collagen [15] and subsequent collagen induced platelet activation and aggregation via GPVI [16]. By NMR analysis and docking simulation, Ono et al. proposed that losartan binds to a GPVI hydrophobic pocket [17]. However, functional studies were performed using losartan concentrations capable of blocking the TXA2-dependent platelet responses to collagen, and there was no direct experimental evidence that losartan inhibited the binding of GPVI to collagen. Interestingly, Taylor et al recently reported that losartan is more potent on GPVI than on TP, the TXA2 receptor [18]. Yet, several questions remain pending with respect to the mechanism by which losartan inhibits GPVI activation by collagen: inhibition of collagen binding to GPVI or of GPVI dimerization/clustering? The clinical relevance of the antiplatelet effect of losartan also needs to be determined. In this study, we addressed these questions and characterized the effect of losartan on collagen-induced platelet activation. Furthermore, we have compared the platelet functions of normotensive patients treated (n = 25) or not (n = 30) by losartan.

## Material and Methods

### Ethics Statement

All blood donors were volunteers who gave their free and informed written consent to this research study, which conforms to the ethical standards of the Declaration of Helsinki. Legal and ethical authorization for research use of collected blood was obtained through a national convention between the French National Institute of Health and Medical Research (Inserm) and the French Blood Institute (EFS) (convention number I/DAJ/C2675).

### Reagents

Losartan potassium, U46619, an analog of TXA2, ADP, thrombin receptor activating peptide (SFLLRN, TRAP), prostaglandin E1 (PGE1), apyrase grade VII, Duolink In Situ Probemakers and detection reagents were purchased from Sigma-Aldrich (St Louis MO). Losartan metabolites EXP3174 (Losartan Carboxylic Acid; 2-Butyl-4-chloro-1-[[2’-(1H-tetrazol-5-yl)[1,1’-biphenyl]-4-yl]methyl]-1H-imidazole-5-carboxylic Acid) and EXP3179 (Losartan carboxaldehyde; 2-Butyl-4-chloro-1-[[2’-(1H-tetrazol-5-yl)[1,1’-biphenyl]-4-yl]methyl]-1H-imidazole-5-carboxaldehyde; DUP 167) obtained from Toronto Research Chemicals (Toronto, Canada) were dissolved in DMSO as recommended by the manufacturer. Losartan and losartan metabolites were diluted in PBS extemporaneously in order that the final DMSO concentration in platelet samples was always less than 0.5%. Fibrillar-type I Horm collagen from equine tendon (Horm collagen) was from Nycomed (Munich, Germany) and FITC conjugated type I- collagen was from Sigma, St Louis MO). The synthetic peptide that contains the α2β1 integrin specific motif GFOGER and collagen related peptide (CRP) that contains repeats of the GPO motif (O being hydroxyproline) specific of GPVI, were obtained from Richard W. Farndale (Cambridge, UK). Recombinant soluble GPVI was obtained as the extracellular domain of human GPVI fused to the Fc domain of human Igs (GPVI-Fc that is dimeric) or fused to a poly-His tag (GPVI-His that is monomeric) [3]. Their K_Dapp_ for collagen were respectively 1.13 ± 0.03 μg mL^-1^ (5 ± 0.2 nmol/L) and 14.7 ± 3.3 μg mL^-1^ (270 ± 54 nmol/L), in agreement with published data [19, 20].

### Antibodies

The anti-human GPVI monoclonal antibodies 3J24and 9E18 and 9O12 were prepared as previously described [21, 22]. 3J24 recognizes GPVI as a monomer as well as in dimeric or multimeric forms whereas 9E18 does not bind to monomeric GPVI but recognizes GPVI oligomers formed of two or more monomers. 9O12 inhibits GPVI binding to collagen and its Fab blocks collagen-induced platelet activation [7]. Horse radish peroxydase (HRP)-coupled polyclonal goat F(ab’)_2_ anti-human IgG and FITC-coupled monoclonal mouse IgG anti-human P-selectin were purchased from Beckman coulter. FITC-coupled IgM antibody PAC-1 was from BD Science.

### Platelet preparation

Blood from healthy volunteers who had taken no medication during the previous two weeks, was drawn into 3.2% sodium citrate or 15% (v/v) trisodium citrate acid—citric—dextrose (Vacutainer system; Beckton Dickinson, Le Pont-de-Clais, France) and centrifuged to obtain platelet rich plasma (PRP). Washed platelets were prepared from PRP as previously reported [23] and resuspended at a final concentration of 2×10^8^ mL^-1^ in platelet reaction buffer (Hepes 5 mM, NaHCO_3_ 12 mM, NaCl 137mM, KCl 2 mM, CaCl_2_ 2 mM, NaH_2_PO_4_ 0.3 mM, MgCl_2_ 1 mM, glucose 5.5 mM, pH 7.4). At the end of the platelet preparation and before adding the agonists, platelets were allowed to recover from PGE1 and apyrase treatments for 20 min at 37°C.

### Platelet aggregation

Washed platelets or platelet-rich plasma (3×10^8^ platelets.mL^-1^) were preincubated with varying concentrations of losartan or reaction buffer for 10 min at 37°C. Collagen type I (1 μg.mL^-1^), U46619 (1 μM), CRP (0.5 μg.mL^-1^), TRAP (20 μM) or ADP (10 μM) were used to stimulate platelets. Platelet aggregation was continuously recorded as changes in light transmission (APACT 4004, Elitech France).

### Flow cytometry

Platelets (2×10^8^.mL^-1^) were incubated with different concentrations of losartan for 10 min at 37°C, before activation by 10 μg.mL^-1^ collagen type I, 1 μg.mL^-1^ CRP, 10 μM ADP, 20 μM TRAP for 20 min at room temperature (RT). Platelets were incubated with FITC-conjugated anti-P-selectin, PAC1 antibody to the activated integrin αIIbβ3, 9E18 and 3J24 antibodies to GPVI or corresponding isotype matched control, for 20 min at RT and then fixed with 2% PFA. Samples were analyzed by flow cytometry using a LSR II cytometer from Becton Dickinson. Alternatively, platelets pretreated or not with 22 μM losartan were incubated with 10 μg.mL^-1^ FITC conjugated collagen type I for 20 min at RT before fixation and analysis.

### Thrombus formation under flow conditions

Flow chambers (Vena8Fluoro+, Cellix, Dublin, Ireland) were coated with 50 μg.mL^-1^ Horm collagen overnight at 4°C and then saturated with 10 mg.mL^-1^ BSA. PPACK-anticoagulated whole blood was labeled with 10 μM Dioc6 for 10 min at RT then incubated or not with 22 μM losartan for 10 min at 37°C. Blood was perfused in the flow chambers for 5 min at a shear rate of 1500 s^-1^. Platelet adhesion was observed under a Zeiss Axiovert 200M inverted microscope and continuously recorded using an AxioCam MRm vers.3 camera. At the end of the perfusion, pictures covering the length of the channel were taken. Quantitative analysis was performed using ImageJ image analysis software.

### Platelet adhesion

Platelet adhesion onto immobilized collagen or the α2β1 integrin specific collagen-like peptide GFOGER was examined in static condition as described [7]. Briefly, Immulon 2HB 96-well plates (Thermo Scientific, MA, USA) were coated with Horm collagen (100 μL, 10 μg.mL^-1^) or GFOGER (100 μL, 20 μg.mL^-1^) overnight at 4°C. After saturation with 10 mg.mL^-1^ BSA in PBS for 2 hours at RT, washed platelets (2×10^8^.mL^-1^) in reaction buffer completed or not with Mg^2+^, treated or not with 22 μM losartan, were incubated into collagen- or GFOGER-coated wells for different times. After washing, the amount of adherent platelets was determined by quantifying alkaline phosphatase activity using p-nitrophenyl phosphate (pNPP).

### Binding of GPVI to collagen

Immulon 2HB 96-well plates were coated with collagen and saturated as described above. 10 μg.mL^-1^ GPVI-His and 0.25 μg.mL^-1^ GPVI-Fc, pretreated with 0, 22, 110, 220 μM losartan, were incubated with collagen for 2 hours at RT. GPVI-His and GPVI-Fc were revealed using HRP-coupled anti-6×His and anti-human IgG antibodies respectively and O-phenylenediamine dihydrochloride (OPD), as described [24].

### Clustering of GPVI

The anti-human GPVI monoclonal antibody 9E18 was conjugated with Duolink PLA probe PLUS and MINUS separately using Duolink In Situ Probemaker kit following the instructions of the manufacturer. Lab-Tek chambers were coated with Horm collagen (200 μL, 10 μg.mL^-1^) overnight at 4°C and saturated with 10 mg.mL^-1^ BSA. Platelets treated or not with 22 μM losartan, and mixed with 5 μg.mL^-1^ of the PLUS-probe-coupled antibody and 5 μg.mL^-1^ MINUS-probe-coupled antibody and then incubated in collagen coated wells for 45 min at 37°C. After removing unbound platelets, adherent platelets were fixed with 4% PFA in PBS. Ligation, amplification and detection with Duolink In Situ Detection Reagents Orange were done according to the manufacturer’s instructions. In this procedure, bright fluorescent dots were observed when two probes are in close proximity. Platelets were labeled with anti-CD41-Alexa Fluo 488. Images from the collagen-contacting surface of platelets were captured using a Zeiss Axiovert 200M inverted microscope equipped with Zen image capture system. Platelet adhesion was measured by quantifying the surface covered by adherent platelets on 30 random fields obtained from 3 independent experiments. Orange Dots associated to platelets were counted as GPVI clusters on the same fields.

### Statistical analysis

All data obtained *in vitro* are expressed as mean+SEM. All pairwise comparisons were made relative to the control condition (samples with losartan are compared to the paired samples without losartan (same platelet donor, same day). Paired t-test and one-way ANOVA followed by a Bonferroni correction for multiple comparisons were performed using GraphPad Prism software (San Diego CA). P values <0.05 were regarded as statistically significant

### *Ex vivo* studies

To determine whether the therapeutic dose of losartan is capable of reducing platelet reactivity *ex vivo*, we performed a blinded analysis of blood samples from patients randomized to treatment by losartan (100 mg losartan per day) *vs* placebo. These patients are suffering from Marfan syndrome and the assay was designed to determine the efficacy of losartan on aortic dilatation (ClinicalTrials.gov Identifier: NCT00763893 published as [25]. The protocol was authorized by the IRB “Comité de Protection des Personnes Île-de-France XI, St Germain en Laye”. All patients provided written informed consent.

The study complies with the Declaration of Helsinki and was approved by the institutional ethics committee. The evaluation of the platelet function performed on 55 patients was an ancillary study of the main trial (300 patients). Platelet-rich plasma isolated from citrate anti-coagulated blood was used to measure platelet aggregation induced by collagen (1 μg.mL-^1^), U46619 (1 μM), ADP (10 μM) and TRAP (20 μM). P-selectin exposure and GPVI dimerization were measured by flow cytometry on resting and activated platelets. After identification of the samples, the two groups were compared using a Wilcoxon rank-sum test along with the confidence interval of the Hodges-Lehmann’s median differences.

## Results

### Losartan specifically inhibits collagen-induced platelet aggregation and activation *in vitro*

**Fig 1:**
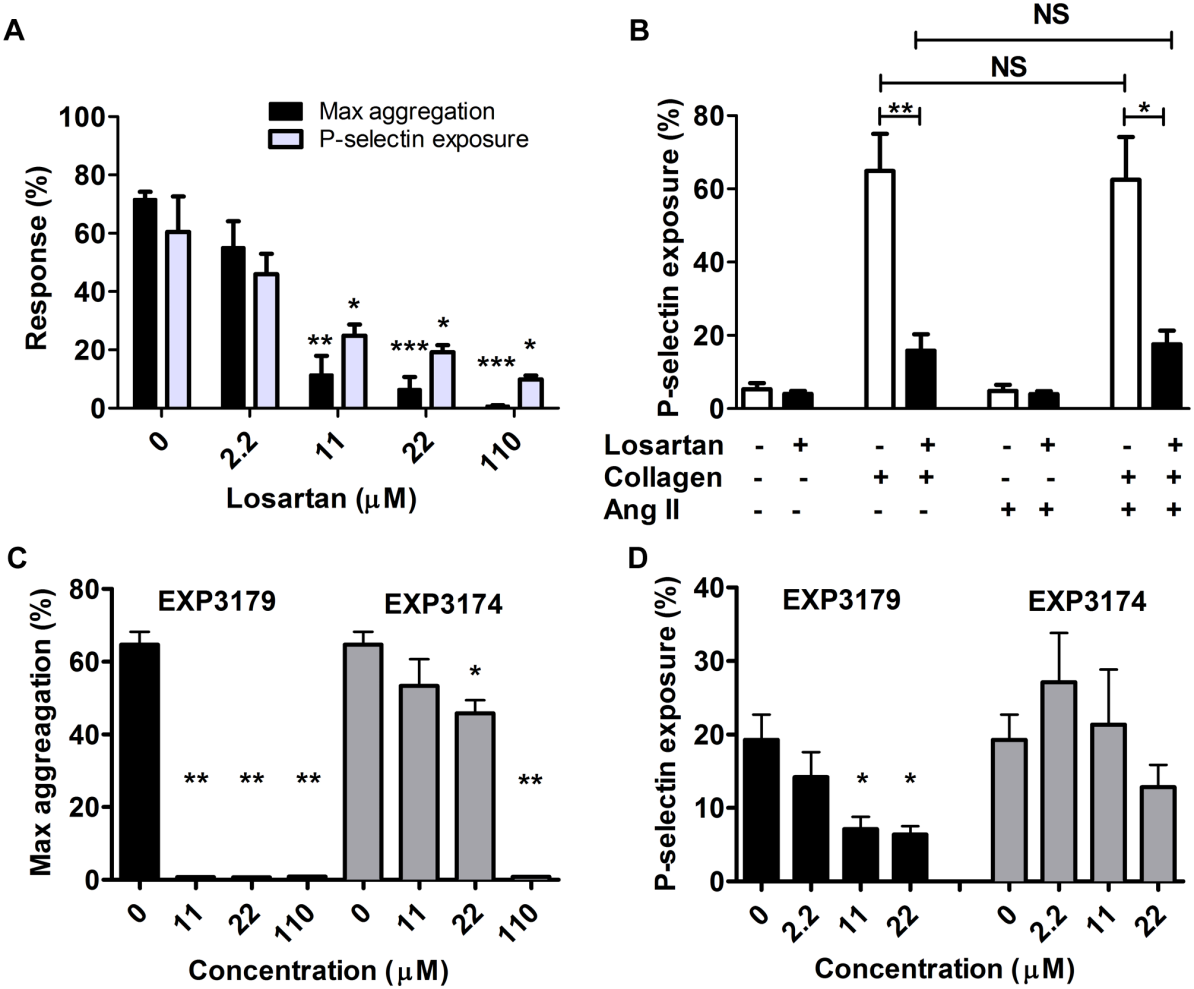
Losartan dose-dependently and specifically inhibits collagen induced platelet activation. Washed human platelets were incubated with losartan (A, B,), EXP3179 or EXP3174 (C, D) for 10 min at 37°C. Platelet aggregation (A, C) and P-selectin exposure (A, B, D) were triggered by collagen 1 μg.mL^-1^ and 10 μg.mL^-1^, respectively. In (B), washed platelets pre-treated with PBS (open bars) or 22 μM losartan (black bars), were incubated or not with 10 nM angiotensin II for 10 min and then activated by 10 μg.mL^-1^ collagen before measuring P-selectin exposure. Maximal aggregation is reported (n = 4 for all doses of losartan,); in (A, B and D), P-selectin exposure was measured by flow cytometry and percentage of positive platelets is reported (n = 5 for all doses of losartan). All the data are mean +SEM of n = 4 experiments (platelet aggregation) or n = 5 experiments (P-selectin exposure). Pairwise comparisons were made relative to the condition without losartan; one-way ANOVA followed by a Bonferroni correction was used for multiple comparisons *P<0.05, **P<0.01, ***P<0.001, NS: not significant.

Losartan dose-dependently inhibited the aggregation of human washed platelets induced by 1 μg.mL^-1^ collagen with an IC50 of 6.5 μM (Fig 1A) in agreement with recently reported data [18]. Furthermore, losartan inhibited the exposure of P-selectin induced by 10 μg.mL^-1^ collagen with an IC50 of 6.3 μM. P-selectin positive platelets reached 60 ± 12% after 10 min incubation with collagen and dropped to 19 ± 2% in the presence of 22 μM losartan (P = 0.022, n = 5) (Fig 1A). In contrast, losartan had no significant effect on TRAP and ADP induced platelet aggregation and P-selectin exposure (data not shown). We examined the potential impact of an interaction of losartan with AT1R but, Ang II neither induced platelet activation nor modified the response to collagen in the absence or presence of losartan, ruling out a role of AT1R in the losartan effect on platelets (Fig 1B).

The inactive metabolite of losartan, EXP3179, was as effective as losartan to inhibit collagen-induced aggregation and P-selectin exposure (Fig 1C and 1D). In contrast, the active metabolite EXP3174 that is 10–40 times more potent in blocking AT1 receptor than losartan [26] had little effect on both collagen—induced platelet aggregation and P-selectin exposure (Fig 1C and 1D) confirming that losartan impacts collagen-induced platelet activation independently of AT1R.

The formation of TXA2 and interaction with its receptor are required for optimal platelet aggregation in response to collagen. A previous study reported that losartan interacted with the platelet receptor of TXA2, TP, and inhibited platelet activation induced by U46619, an analog of TXA2 [13]. However, in agreement with Taylor et al., we have observed that losartan dose-dependently inhibited platelet aggregation induced by the TXA2 analog U46619 with an IC50 of 40 μM that is 6–7 times higher than the IC50 measured on collagen-induced activation (data not shown). Losartan 22 μM did not significantly reduce U46619-induced platelet aggregation, while the same concentration of losartan inhibited collagen-induced platelet aggregation by 80%. Together these data indicated that losartan, at least at low doses (≤22 μM), inhibited collagen induced platelet aggregation independently of TP. The following experiments were performed using losartan at 22 μM.

### Losartan reduces thrombus formation on immobilized collagen under flow conditions

**Fig 2:**
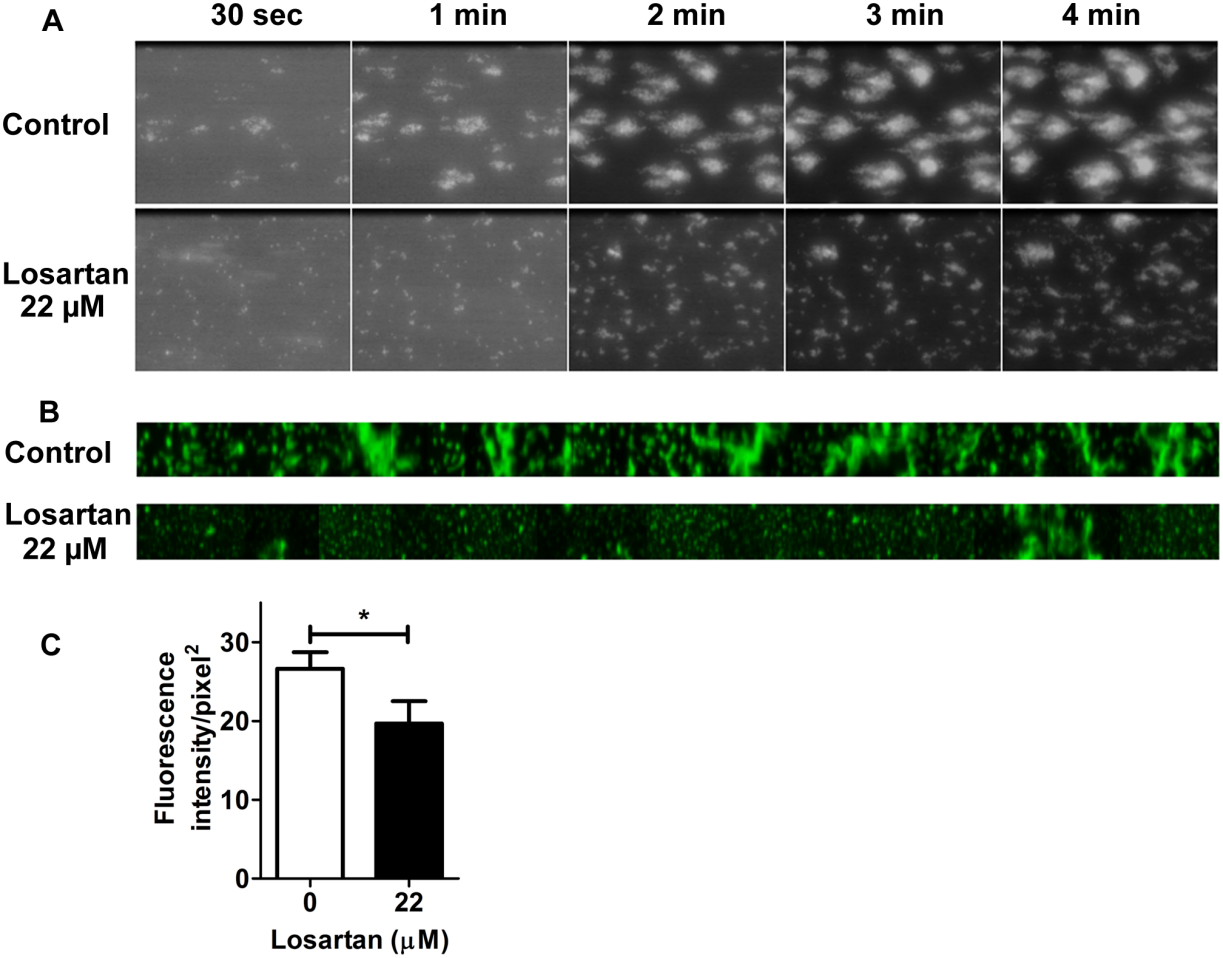
Losartan reduces thrombus formation on immobilized collagen under flow condition *in vitro*. Flow chambers were coated with 50 μg.mL^-1^ Horm collagen overnight at 4°C. PPACK-anticoagulated whole blood was labeled with 10 μM DiOC6 for 10 min at RT then incubated or not with 22 μM losartan for 10 min at 37°C. Blood was perfused in the flow chambers for 5 min at a shear rate of 1500 s^-1^ and platelet adhesion was continuously recorded. Images captured at different times after the beginning of the perfusion (A) and of the full length of chambers after 5 min perfusion (B), are from one representative experiment out of three. Quantitative analysis of the mean fluorescence intensity on the whole chamber (C). Data are the mean + SEM of three independent experiments. * *P*<0.05

The effect of losartan on thrombus formation was analyzed *in vitro* by perfusing anticoagulated whole blood supplemented or not with losartan 22 μM in collagen-coated flow chambers at a shear rate of 1500 s^-1^. As shown in Fig 2A, losartan did not prevent the interaction of isolated platelets with collagen but it decreased the formation of large platelet aggregates. This was confirmed by the quantification of the platelet fluorescence in the flow chamber at the end of the experiment (Fig 2B and 2C).

### Different effects of losartan on platelet activation by collagen related peptides specific of the integrin α2β1 (GFOGER) or of GPVI (GPO)

**Fig 3:**
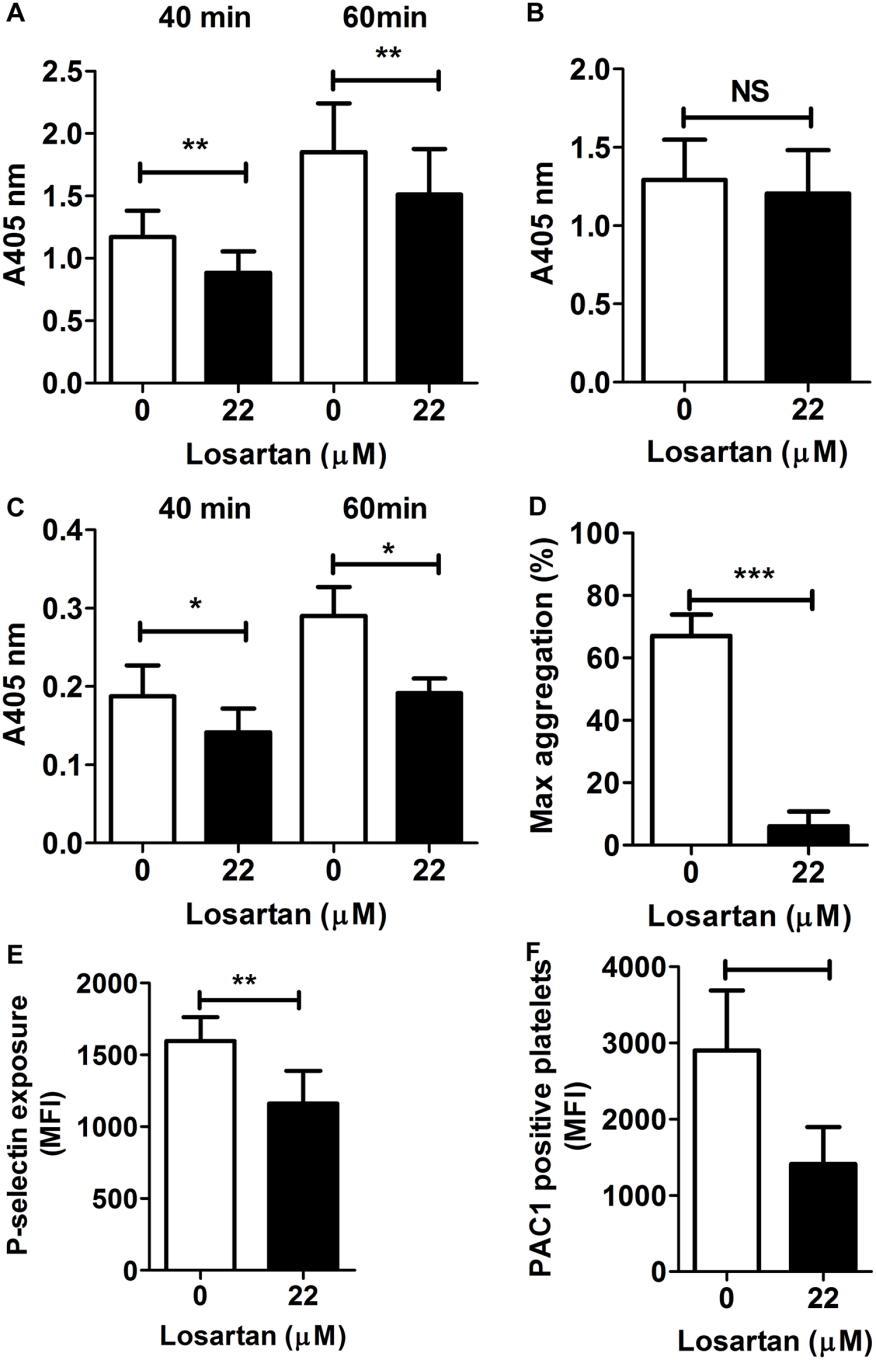
Losartan inhibits collagen induced platelet responses dependent on GPVI and independent on α2β1. Washed platelets in Mg^2+^- and Ca^2+^- free reaction buffer (A) or normal reaction buffer (B-F) were pre-treated with or without 22 μM losartan for 10 min at 37°C. (A) Platelets were incubated in collagen-coated microtiter wells for 40 min and 60 min, respectively. Platelet adhesion was measured by quantifying alkaline phosphatase activity. Data are mean + SEM from 5 independent experiments. (B) Platelets were incubated in GFOGER-coated microtiter wells for 60 min, and adhesion was measured as in (A). Data are mean + SEM from 5 independent experiments. (C) Platelets were incubated in CRP-coated microtiter wells for 40 min and 60 min, and adhesion was measured as in (A). Data are mean + SEM from 6 independent experiments. (D) Platelet aggregation was induced by CRP (0.5 μg.mL^-1^) and was recorded. Data are expressed as mean + SEM of maximal aggregation from 7 independent experiments. (E and F) P-selectin exposure and αIIbβ3 activation were measured by flow cytometry on CRP-activated platelets. Data are expressed as the median fluorescence intensity (MFI) and are from 6 independent experiments. *P<0.05, **P<0.01, NS: not significant.

Since both the α2β1 integrin and GPVI contribute to platelet adhesion on collagen and subsequent activation, we investigated its effect using conditions specific for each receptor. Divalent cations are required for α2β1 but not GPVI interaction with collagen [27]. Losartan inhibited platelet adhesion to collagen in static conditions even in the absence of Mg^2+^ and Ca^2+^ suggesting that its inhibitory effect was independent from α2β1 (Fig 3A). In addition, losartan had no effect on platelet adhesion onto immobilized GFOGER, the collagen motif that specifically binds to α2 I-domain [28] (Fig 3B). In contrast, losartan inhibited platelet activation by GPVI-specific CRP [29] as indicated by a decrease in platelet adhesion (Fig 3C), platelet aggregation (Fig 3D), P-selectin exposure (Fig 3E) and binding of PAC-1 (Fig 3F), a marker of activation of the integrin αIIbβ3.

### Losartan reduces the binding of collagen to platelets

**Fig 4:**
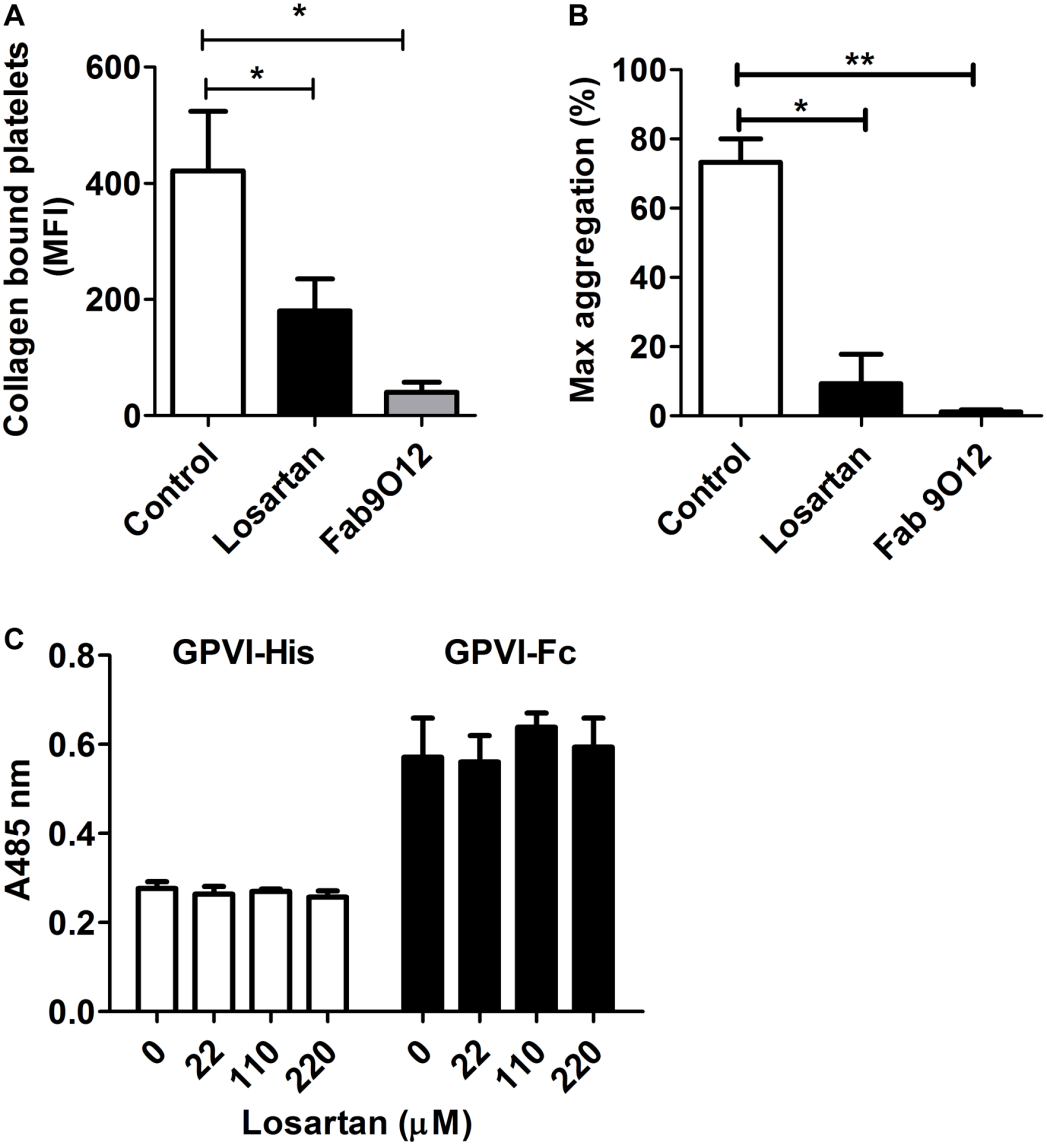
Losartan does not compete with collagen for binding to GPVI. (A) Washed platelets were treated with PBS or 22 μM losartan or 50 μg.mL^-1^ Fab 9O12 for 10 min at 37°C. FITC-coupled collagen (10 μg.mL^-1^) was added and samples were incubated for 20 min at RT and analyzed by flow cytometry. Data are mean + SEM of MFI from 5 independent experiments. (B) The aggregation of control, losartan- or Fab 9O12-treated platelets was triggered by FITC-coupled collagen (1 μg.mL^-1^). Data are expressed as maximal aggregation. (C) GPVI-Fc (0.25 μg.mL^-1^) and GPVI-His (10 μg.mL^-1^) preincubated with increasing concentrations of losartan were added to collagen-coated microtiter wells for 2 h at RT. Bound GPVI was detected using HRP-coupled anti-6×His monoclonal antibody and anti-human IgG, respectively. Data are expressed as mean + SEM from 3 independent experiments.

FITC-conjugated type I collagen was used to further analyze the effect of losartan (Fig 4A). FITC-collagen, at 1 μg.mL^-1^, induced the aggregation of washed platelets that was fully inhibited by the GPVI-blocking Fab 9O12 (Fig 4B). Furthermore, analyzing the binding of FITC-collagen to platelets by flow cytometry showed it was fully inhibited by the Fab 9O12. Finally, a significant reduction in the binding of FITC-collagen to losartan-treated compared with untreated platelets was observed. Together these data indicated that FITC-collagen is capable to activate platelets at least via GPVI and that losartan impairs collagen interaction with GPVI.

### Losartan does not compete with collagen for binding to GPVI

A previous study [17] and our preceding data suggesting that losartan could bind to GPVI, we investigated whether it was capable of blocking the binding of purified GPVI to collagen. For this purpose we used GPVI-Fc, that is dimeric and of high affinity for collagen, and GPVI-His, that is monomeric and of low affinity for collagen [3, 30], at concentrations of 0.25 μg.mL^-1^ and 10 μg.mL^-1^ respectively. Pre-incubation of soluble GPVI with increasing concentrations of losartan up to 220 μM had no effect on the binding of either GPVI-Fc or GPVI-His to immobilized collagen (Fig 4C). These data indicated that losartan is not capable of directly blocking the binding of GPVI to collagen, and thus suggested a mechanism of action different from simple competition.

### Losartan inhibits collagen induced clustering of GPVI on platelets

In agreement with others [18] we have observed that losartan (22 μM) delayed collagen-triggered protein tyrosine phosphorylation and reduced its extent (data not shown). The phosphorylation of the GPVI signaling subunit FcRγ examined by immunoprecipitation, was reduced in the samples of losartan-treated platelets compared to control samples confirming that that losartan impacts the initial phase of the GPVI signaling pathway.

**Fig 5:**
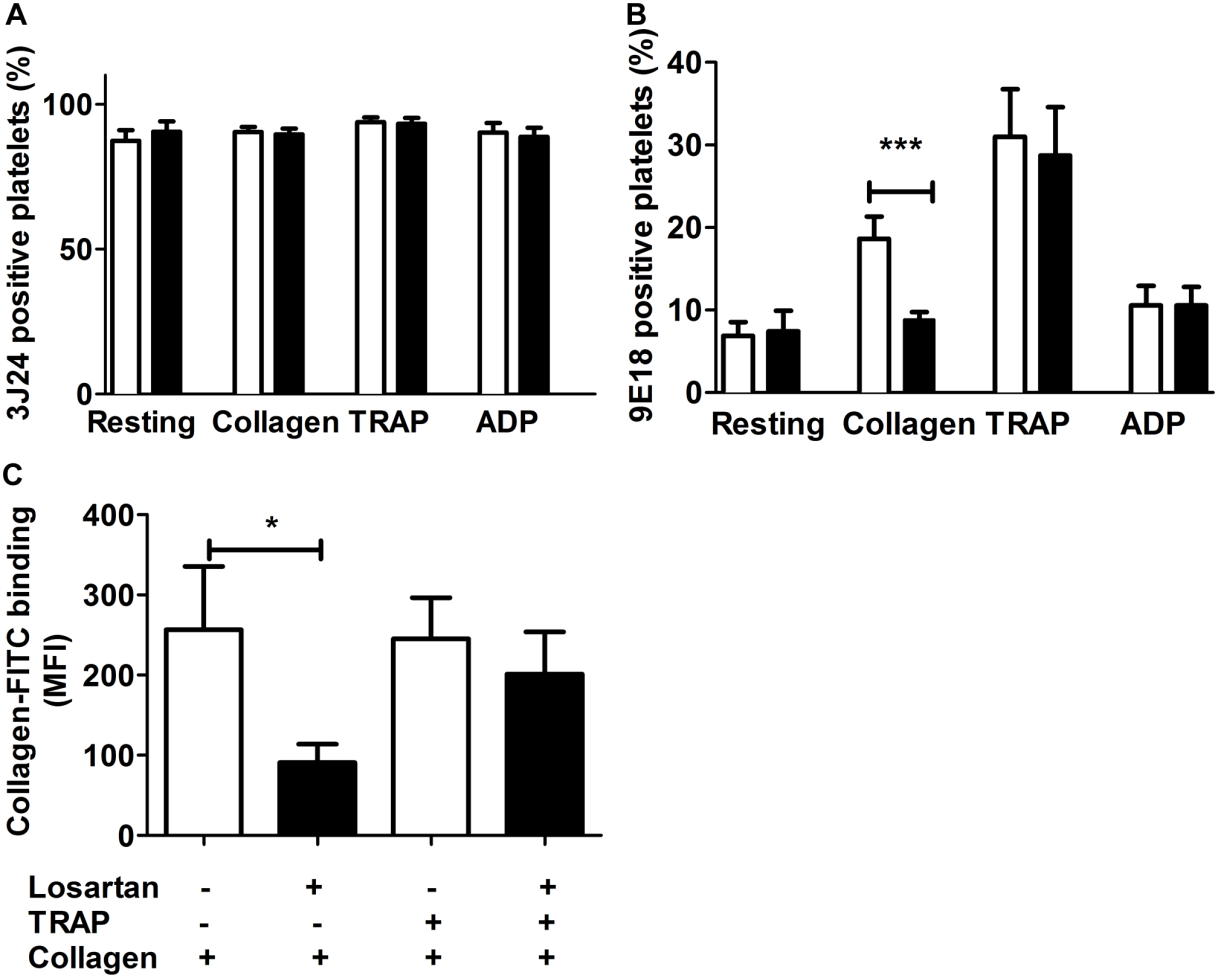
Losartan inhibits collagen induced clustering of GPVI but not ADP- or TRAP-induced GPVI dimerization. Washed platelets preincubated with PBS or losartan (22 μM), were stimulated with collagen (10 μg.mL^-1^), TRAP (20 μM) or ADP (10 μM) for 20 min. (A) GPVI expression and (B) GPVI dimerization were measured by flow cytometry using FITC-coupled 3J24 and 9E18, respectively. Results (mean + SEM) are from 5 independent experiments. (C) Washed platelets pre-treated with PBS or 22 μM losartan and pre-stimulated or not with TRAP (20 μM) were incubated with FITC-coupled collagen (10 μg.mL^-1^). Bound collagen was analyzed by flow cytometry. PBS: open bars; losartan: black bars. Data are mean + SEM from 7 independent experiments. * *P*<0.05, *** *P*<0.001.

Now, activation of GPVI results from the clustering of the receptor [31, 32]. While GPVI is mainly monomeric on resting platelets, dimers are formed in response to ADP, TRAP or vWF. However these dimers are not competent to signal [3]. In contrast, collagen-induced GPVI clustering results in the phosphorylation of FcRγ. We thus examined the effect of losartan on GPVI dimerization and clustering using the monoclonal antibody 9E18 that recognizes GPVI oligomers formed of at least 2 monomers. Losartan modified neither the level of GPVI (Fig 5A) nor the binding of 9E18 to ADP- or TRAP-treated platelets (Fig 5B) indicating that losartan does not inhibit GPVI dimerization triggered by inside-out signaling. In contrast, losartan significantly inhibited the binding of 9E18 to collagen-activated platelets. Furthermore, losartan had no significant effect on the binding of FITC-collagen to TRAP-treated platelets, that is in contrast to the strong inhibition observed when platelets were not pre-activated by TRAP (Fig 5C). Together these data indicated that losartan neither inhibited the formation of GPVI dimers nor collagen binding to GPVI dimers but rather blocked GPVI clustering by collagen and downstream signaling.

**Fig 6:**
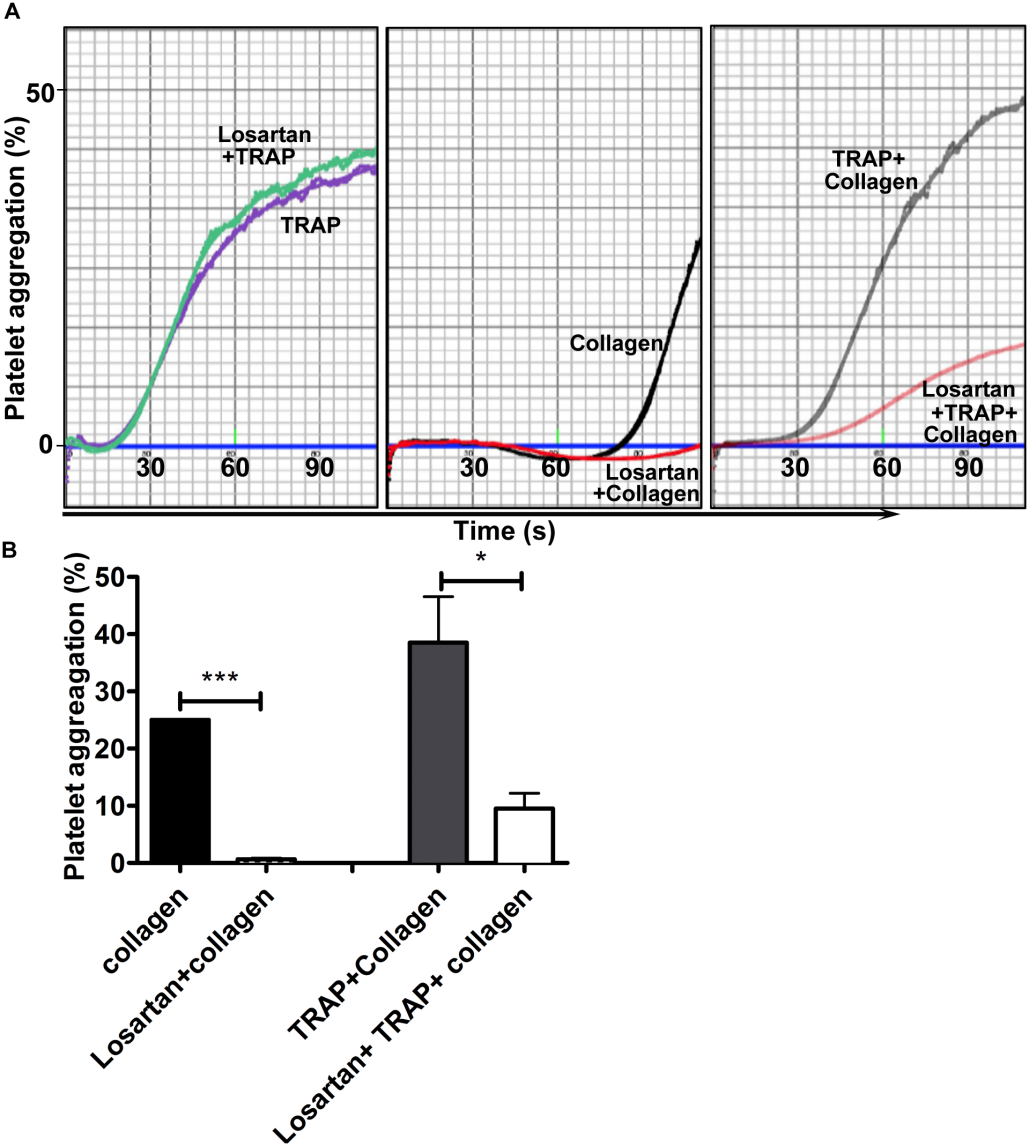
Losartan inhibits collagen induced platelet activation even when GPVI dimers are preformed. Washed platelets were preincubated with PBS or losartan (22 μM) for 10 min at 37°C. Buffer or TRAP (10 μM) were added to the samples and the incubation continued without stirring for 10 min. Collagen (1 μg.mL^-1^) was then added, stirring started and aggregation recorded. (A) Representative aggregation curves. (B) When the aggregation reached 25% in control condition (preincubation with PBS, no losartan), the extent of aggregation in other conditions was measured. Data are represented as bar charts. (Mean + SEM of 3 experiments)

To test this hypothesis, collagen-induced aggregation was measured using platelets that have been pretreated or not with TRAP in the absence of stirring (Fig 6) to trigger the formation of GPVI dimers as shown above. Pretreatment with TRAP increased the rate of platelet activation by collagen in agreement with the TRAP-induced formation of GPVI dimers. However, losartan reduced the aggregation response triggered by collagen even when platelets had been pretreated by TRAP. These data further indicate that losartan impacted the clustering of GPVI by fibrillar collagen.

**Fig 7:**
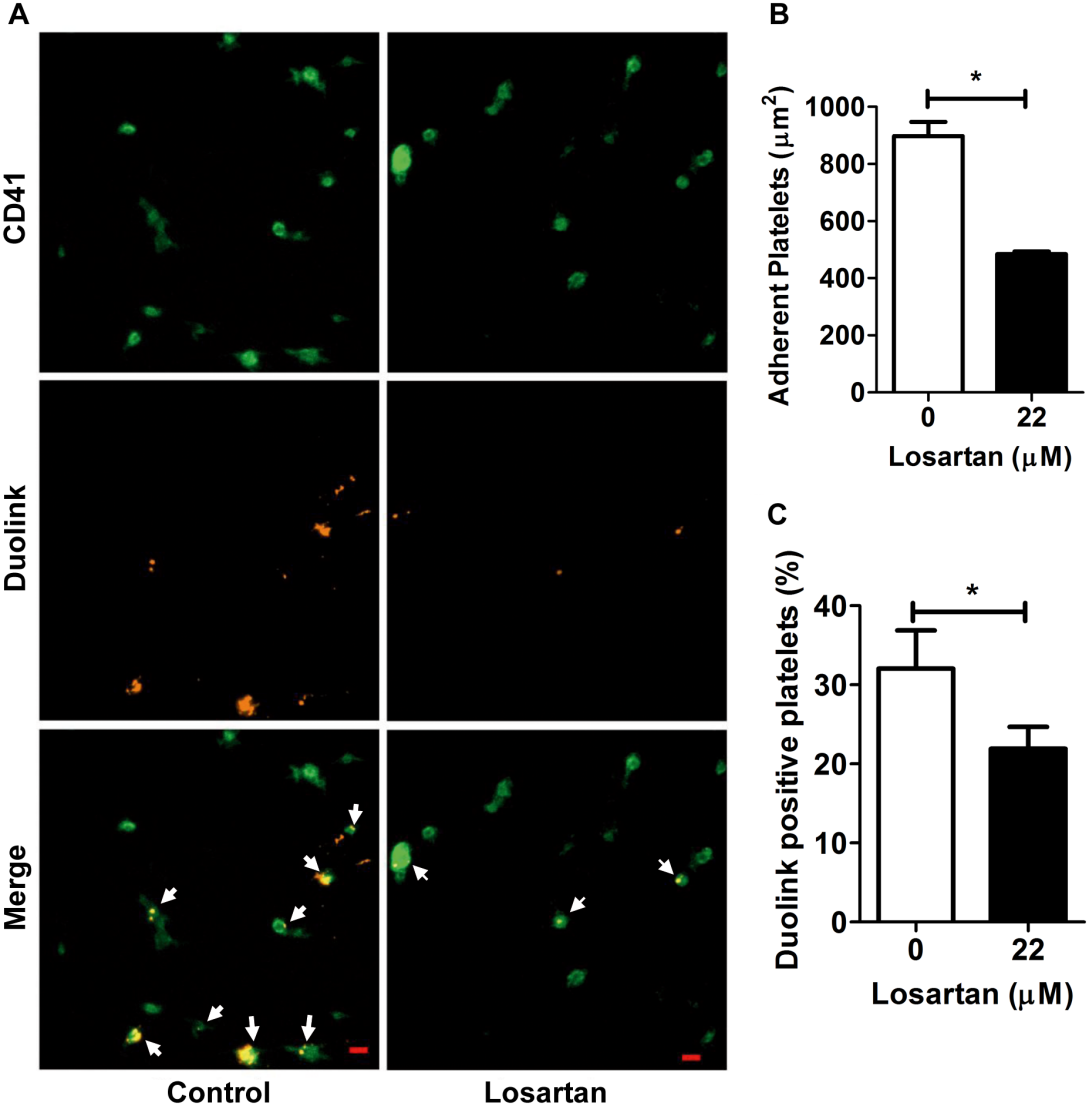
Duolink imaging of collagen-induced GPVI clustering and its inhibition by losartan. (A) Washed platelets pretreated with PBS or losartan (22 μM) were incubated with 5 μg.mL^-1^ 9E18-MINUS and 5 μg.mL^-1^ 9E18-PLUS and added to collagen-coated Lab-Tek chambers for 45 min at 37°C. Bound platelets were fixed with 4% PFA and stained with 40 μg.mL^-1^ anti-CD41 alexa fluo 488 (Green) before ligation and the MINUS and PLUS oligonucleotides and amplification. PLA dots (Orange) are only generated if 9E18-MINUS and—PLUS are in close proximity (<40 nm). Numerous and large GPVI clusters are observed in control conditions while only few spots are visible in the presence of losartan. (B) Quantitative analysis of adherent platelets (green). Data are expressed as the area covered by platelets and the mean + SEM of n = 30 random fields from 3 independent experiments. Scale bars: 5 μm. *P<0.05. (C) Quantitative analysis of platelets GPVI clustering as determined by measuring the proportion of Duolink positive platelets on the same fields.

To further assess the clustering of GPVI, we used the Duolink in Situ labelling procedure with 9E18 coupled to minus and plus-oligonucleotides. In these conditions no signal could be detected unless two or more GPVI dimers become adjacent and form clusters (n≥4 GPVI) allowing amplification of the signal. In fact, in the absence of losartan, platelet interaction with collagen resulted in the formation of platelet associated fluorescent spots indicating the clustering of GPVI dimers (Fig 7A). As expected losartan reduced platelet adhesion to collagen (Fig 7B) and, very interestingly, the proportion of Duolink-positive signals was significantly decreased on adherent platelets (Fig 7C), confirming that the clustering of GPVI was impaired by losartan.

### Platelets from losartan-treated patient do not exhibit a functional defect *in vivo*

Fifty-five patients with Marfan syndrome were analyzed, 25 treated by losartan and 30 receiving the placebo. Collagen- (1 μg.mL^-1^) and U46619- (1 μM) induced platelet aggregation did not show statistically significant differences between the two groups (Table 1). The extent of aggregation was similar as well as the lag that is the first parameter to be changed (increased) at a moderate inhibition. The levels of P-selectin exposure and GPVI dimerization were low on non-activated platelets from the two groups and not significantly different (Table 1) whereas the levels of GPVI expression as measured using the 3J24 antibody were not different between the two groups of patients and between Marfan syndrome patients and healthy controls [83.37 ± 12.8% *vs* 87.9 ± 11.6% (mean ± SD)]. In agreement with previous studies [3], the levels of 9E18 binding to resting platelets was lower when measured in PRP than on washed platelets (Fig 5B) Both P-selectin exposure and GPVI dimerization increased in response to collagen or U46619 and were not statistically different between the two groups. These data indicated that the therapeutic dose of losartan received by these normotensive patients did not impact, *ex vivo*, the responses of platelets to collagen.

**Table 1:** Platelet functions of patients treated by losartan or placebo.

	Placebo n = 30	Losartan n = 25	95% Confidence Interval of the difference	*p*-value
Intensity (%) collagen	83.5 [20.6–99.9]	80.7 [20.2–93.7]	[-6.97; 3.79]	0.51
Intensity (%) U46619	83.1 [53.1–109.5]	84.5 [65.7–104.2]	[-4.23; 4.75]	0.89
Lag time (s) collagen	56.4 [21.2–98.6]	58.8 [16.4–119.2]	[-9.2; 12]	0.94
Lag time (s) U46619	11 [8–11.4]	11 [8.6–15.2]	[-0.2; 0.6]	0.19
P-sel (%) resting	2.07 [1.1–7.3]	2 [0.55–6.7]	[-8; 1.5]	0.3
P-sel (%) collagen	7.82 [2.05–73.9]	5.25 [0.85–6.7]	[-11.7; 0.6]	0.16
3J24 (%) resting	91.47 [63.85–98]	83.4 [44.25–98.6]	[-13.55; 0.95]	0.17
9E18 (%) resting	1.1 [0.2–7]	1.35 [0.4–5.35]	[-0.5; 0.5]	0.83
9E18 (%) collagen	5.05 [1.2–31.2]	3.6 [0.95–14.15]	[-4.05; 0.35]	0.17

Platelet aggregation was measured on PRP, the maximal intensity and lag lime are reported; platelet P-selectin exposure and GPVI dimerization (9E18 binding) were measured by flow cytometry in resting conditions or after activation by collagen. Statistical analysis (Wilcoxon rank-sum test) showed no difference between the two groups. Data are expressed as median [ranges] except for CI and *p*-values.

## Discussion

GPVI is recognized as an attractive target for developing new efficient and safe antiplatelet drugs. The possibility that small molecules already used in therapy could block GPVI raises interest. Losartan, an AT1-receptor antagonist used for the treatment of hypertension, was reported to inhibit collagen induced platelet adhesion, activation and aggregation *in vitro* and *in vivo* [15, 16, 33]. The recent data of Taylor et al. [18] have clarified the effect of losartan on GPVI and TP respectively showing that losartan is 5 to 10 times more potent on GPVI than on TP. Our work provides new insight into the mechanism of losartan effect on platelets. The major findings are that losartan inhibits collagen-induced platelet activation *in vitro* via an original mechanism that is the inhibition of GPVI clustering by collagen and that the administration of a therapeutic dose of losartan to normotensive patients had no measurable biological effect on platelet reactivity.

First, we confirmed that losartan and its inactive metabolite EXP3179 inhibit collagen-triggered aggregation and activation of platelets either washed or in PRP, which is consistent with previously reported observations [16]. Furthermore we demonstrated the capacity of losartan in whole blood to inhibit thrombus formation on collagen in arterial flow conditions. Ang II has been reported to induce and potentiate the shape change of human platelets and this change was inhibited by losartan [34] but in another study, only very low concentrations of Ang II appeared active on platelets [35]. Here, the observations that collagen-induced platelet activation was neither inhibited by EXP3174 that is 10–40 times more potent in blocking AT1 receptor than losartan [26] nor promoted by Ang II are in agreement with the study of Grothusen et al. [16] and rule out a role of AT1R in collagen-induced platelet activation and its inhibition by losartan.

The release of ADP and TXA2 plays an important role in the multistep process of collagen induced platelet aggregation [36]. Losartan was reported to inhibit U46619-induced platelet aggregation via a competitive inhibition of TP [13, 14]. In fact, as Taylor we observed that losartan has a lower capacity to inhibit U46619- than collagen-induced platelet activation as indicated by the 6-fold higher IC50. Thus, low concentrations of losartan (≤22 μM) inhibit collagen-induced platelet activation largely independently of its effect on TP. In contrast, in studies using high doses of losartan, up to 500 μM, inhibition of TP should be considered important in the inhibition of collagen-induced platelet aggregation [16].

Platelet GPVI and α2β1 integrin both directly interact with collagen and are involved in platelet activation and adhesion onto collagen [1]. GFOGER peptides contain the minimal recognition sequence for the collagen-binding α2I-domain [28] while binding of collagen to α2β1 on platelets requires millimolar concentrations of Mg^2+^ and Ca^2+^ [27]. The observations that losartan did not impact platelet adhesion to GFOGER and reduced platelet adhesion to collagen even in the absence of Mg^2+^ and Ca^2+^ ruled out α2β1 as the target of losartan.

In contrast, losartan inhibited platelet activation by GPVI-specific CRP in favor of GPVI-dependent mechanism of action in agreement with docking studies [17]. This was further indicated using FITC-conjugated collagen that bound and activated platelets in a GPVI-dependent manner: losartan reduced the binding of FITC-collagen to platelets and FITC-collagen-induced platelet aggregation. These data together with the observation made by Taylor et al [18] and by us that losartan inhibited collagen-induced phosphorylation of FcRγ, the very initial step of signaling downstream of GPVI were in favor of the proposal that losartan occupied the binding site on GPVI collagen. Determining whether losartan behaved as a competitive or non-competitive inhibitor of collagen interaction with GPVI on platelets proved complicated. In fact, the interaction of collagen or CRP with GPVI is not one to one, since a single collagen or CRP molecule binds to several GPVI copies on the same or different platelets. In addition, at a dose higher than 22 μM the effect of losartan on collagen-induced platelet activation is not pure since it also inhibits TPR. Yet, competition between losartan and collagen for binding to GPVI was ruled out by the fact that losartan up to 220 μM did not impact the binding of recombinant GPVI to collagen. This was true for monomeric GPVI, the predominant conformation on resting platelets, and for dimeric GPVI, that has a high affinity for collagen and inhibits collagen-induced platelet activation by a competitive mechanism [20].

We then hypothesized that losartan could affect GPVI clustering, the event that triggers the signaling cascade. First, the effect of losartan on GPVI dimerization was analyzed taking advantage of the ability of TRAP and ADP to induce inside-out signals leading to the assembly of GPVI dimers [3]. Losartan was without effect on the binding of the 9E18 antibody to ADP- or TRAP-activated platelets, indicating that it does not block the homotypic interactions required for dimerization. Interestingly, the binding of FITC-collagen to platelets expressing preformed GPVI dimers was not inhibited by losartan, confirming that losartan does not compete with collagen for binding to GPVI dimers and suggesting that losartan blocks the clustering of GPVI by collagen, a step required for downstream signaling. Indeed, when GPVI dimerization was triggered by TRAP allowing the binding of collagen to GPVI in the presence of losartan, platelet aggregation in response to collagen was still inhibited by losartan.

To further investigate the possibility that losartan blocked collagen-induced GPVI clustering, we took advantage of the Duolink strategy. The 9E18 antibody was coupled to two complementary oligonucleotides, the amplification of which requires that at least two GPVI dimers come in close proximity, thus signaling the formation of GPVI clusters. A technical limit of this method is that it requires to be performed on adherent platelets. Despite losartan reduced platelet adhesion, a significant reduction of platelet-associated positive signals was though observed providing the direct proof of losartan’s capacity to inhibit collagen-induced GPVI clustering.

**Fig 8:**
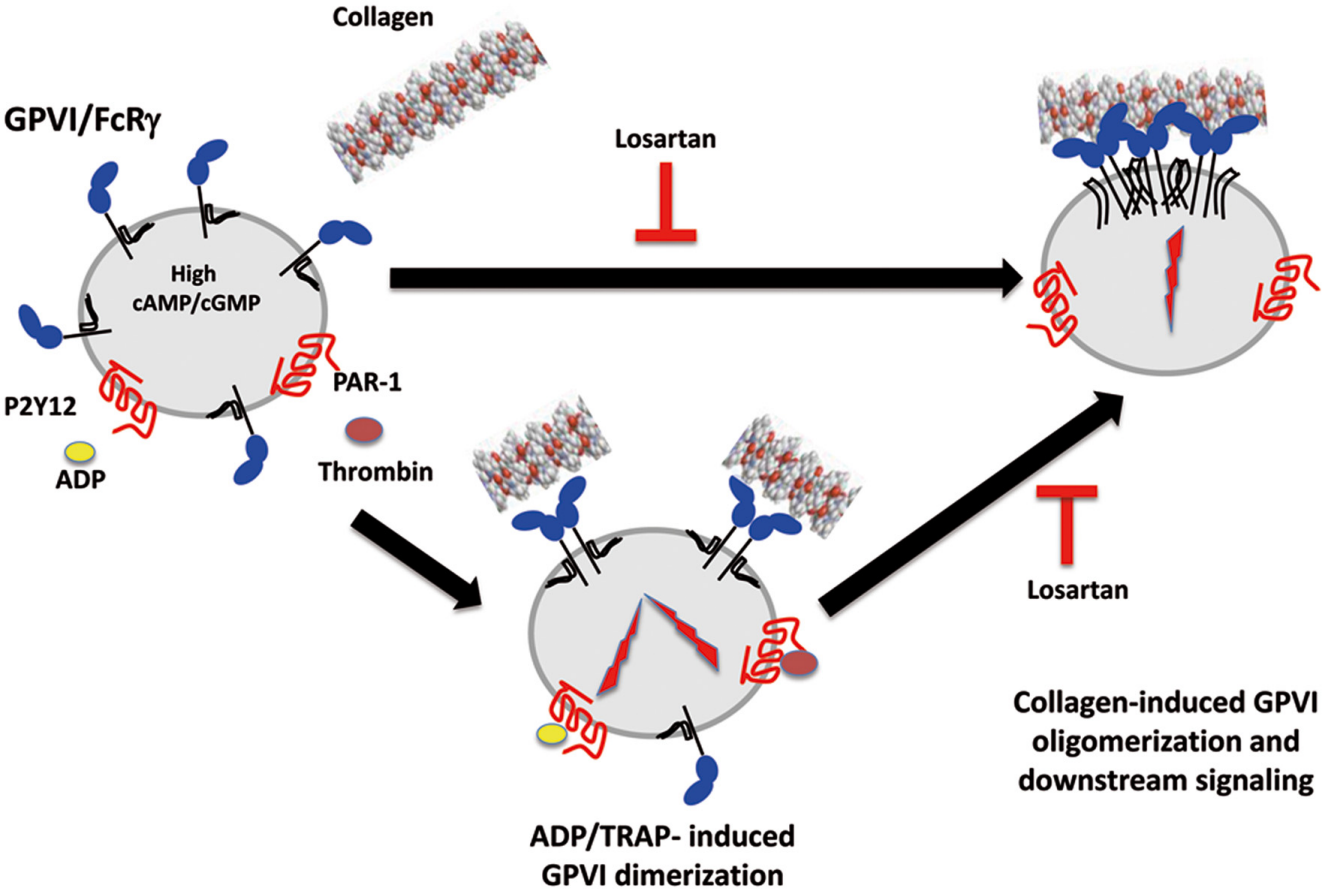
Schematic representation of the inhibitory effect of losartan on collagen-induced platelet activation. Losartan does not impact GPVI dimerization in response to ADP and TRAP but blocks GPVI clustering by collagen and subsequent platelet responses.

Aware of the limitations of current methodologies even if innovative, the hypothesis that losartan inhibits the clustering of GPVI by collagen appears so far as the best supported by the data. The mechanism of action of losartan is thus schematized on Fig 8. When platelets are stimulated by collagen, GPVI clustering triggers signaling [31, 32] and it is inhibited by losartan. When ADP or TRAP stimulates platelets, GPVI dimerization in triggered by an inside-out signaling and collagen binds to GPVI dimers even in the presence of losartan, but clustering and downstream signaling is inhibited by losartan.

The limit of our study is that the molecular events involved in GPVI clustering and its inhibition by losartan are not identified. NO production by platelets could be involved, since losartan-induced NO production by platelets was previously shown to be related to the inhibition of platelet adhesion to collagen [33]. This is in agreement with our previous observation that NO donors prevent GPVI dimerization [3]. However, while EXP3174 was reported to also induce NO production [33], we and others [16] showed it did not inhibit platelet activation by collagen. Another hypothesis relates to a more general effect of losartan on cell surface receptors. Indeed, even if we excluded a direct relation between the inhibition of TP and of GPVI by losartan, it is interesting to note that losartan at supra-therapeutic concentrations inhibits the functions of other receptors such as TP, the stimulation of which was previously shown to provoke the clustering of the CX3CL1 receptor [37]. Recently, biophysical studies showed a strong interaction of losartan with membranes suggesting a role of membranes in losartan’s pathway toward AT1R [38]. However, the authors showed that valsartan adopts a similar position although this drug had no effect on GPVI signaling [18] suggesting that the interaction of sartans with the plasma membrane does not account for the inhibitory effect on GPVI.

Losartan and its metabolite EXP3179 are capable of inhibiting platelet activation by collagen *in vitro* with an IC50 of <7 μM higher than, but not far from the plasma concentration of losartan in treated patients, reported to reach 1 μM after one oral dose [39]. It was proposed that the antiplatelet properties of losartan might contribute to its beneficial effect on the risk of thrombosis [16]. Indeed, losartan was recently reported to prolong the occlusion time in a mouse model of carotid injury [40]. Moreover, the therapeutic use of losartan goes beyond hypertension: Losartan is used for its protective effect against dilation in aortic aneurysm [41–43] and proteinuria in glomerulonephritis [44, 45]; it is proposed to be anti-fibrotic, with some studies supporting the hypothesis of a cross-talk between AT1R and TGF-β1 [46]. It is worth noting that platelet activation may be associated with aneurysm dilatation [47] and glomerulonephritis, in which GPVI was shown to contribute [48]. Thanks to a clinical trial aimed to determine the efficacy of losartan on aortic dilatation in patients with Marfan syndrome, we have performed a blinded assay analyzing platelet functions of patients who received losartan or placebo. Light transmission aggregometry and flow cytometry failed to demonstrate any statistically significant difference in the platelet reactivity of losartan- *vs* placebo-treated patients. Our data differs from the recent study of Sakamoto et al [49] who observed a reduced platelet aggregability in response to ADP in patients (n = 20) suffering from atrial fibrillation and treated with 100 mg losartan daily. However, we and others did not observe any effect of losartan on ADP-induced platelet aggregation. It is thus highly probable that the lower platelet aggregation reported by Sakamoto in losartan-treated patients relies on the beneficial effect of losartan on hypertension and risk factors of thrombosis such as evidenced by the decline in tissue factor and PAI-1. In contrast our data indicate that in normotensive patients, the therapeutic daily dose of 100 mg of losartan does achieve a biological anti-platelet effect. This means that losartan does not fulfill the requirements expected for a therapeutic antagonist of GPVI.

In conclusion, the antiplatelet effect of losartan observed *in vitro* is unlikely to reach efficacy in patients receiving a therapeutic dose. However, our data provide evidence for a novel mechanism for inhibiting GPVI function that is based on the inhibition of GPVI clustering. Losartan could therefore be considered as a model for developing new antithrombotic small inhibitors of GPVI of potential therapeutic interest.

## Supporting Information

S1 Raw Data (XLSX)
